# Clinical manifestation and management of immune checkpoint inhibitor‐associated cardiotoxicity

**DOI:** 10.1111/1759-7714.13250

**Published:** 2019-12-17

**Authors:** Xiaoxiao Guo, Hanping Wang, Jiaxin Zhou, Yue Li, Lian Duan, Xiaoyan Si, Li Zhang, Ligang Fang, Li Zhang

**Affiliations:** ^1^ Department of Cardiology, Peking Union Medical College Hospital Peking Union Medical College and Chinese Academy of Medical Sciences Beijing China; ^2^ Department of Respiratory Medicine Peking Union Medical College Hospital, Peking Union Medical College and Chinese Academy of Medical Sciences Beijing China; ^3^ Department of Rheumatism and Immunology Peking Union Medical College Hospital, Peking Union Medical College and Chinese Academy of Medical Sciences Beijing China; ^4^ Department of Gastroenterology Peking Union Medical College Hospital, Peking Union Medical College and Chinese Academy of Medical Sciences Beijing China; ^5^ Department of Endocrinology Peking Union Medical College Hospital, Peking Union Medical College and Chinese Academy of Medical Sciences Beijing China; ^6^ Department of Clinical Laboratory Peking Union Medical College Hospital, Peking Union Medical College and Chinese Academy of Medical Sciences Beijing China

**Keywords:** Cardiotoxicity, immune checkpoint inhibitors, myocarditis

## Abstract

Immune checkpoint inhibitors (ICIs) targeting programmed death‐1 (PD‐1), its ligand (PD‐L1), and cytotoxic T‐lymphocyte‐associated antigen 4 (CTLA4) have revolutionized cancer treatment by recovering the attack of T lymphocytes on the malignant cells. They have improved clinical outcomes dramatically in multiple types of advanced‐stage malignancies. Even though the tolerance and safety profiles are generally good, it has been widely reported that ICIs can cause severe or fatal immune‐related adverse events (irAEs), since the activated T lymphocytes are not specific for tumor cells. Cardiac irAEs appear to occur less frequently than irAEs in other organ systems but are notorious for high mortality. Here, we aim to identify and characterize the ICI‐associated cardiotoxicity and summarize the optional diagnosis and treatment strategies.

## Introduction

The immune system is a systemic barrier for the human body to monitor the development of malignant tumors. Tumor cells evade immune surveillance by altering their biological characteristics such as overexpressing checkpoint proteins that prevent immune cells from killing them. In recent years, the role of immune factors in tumorigenesis and metastasis has received more attention. Immune checkpoint inhibitors (ICIs) have become the most promising type of immunotherapy in the field of anticancer research due to their remarkable clinical benefit.^1^ In particular, it has shown great strength in the treatment of advanced‐stage malignancies such as melanoma, non‐small‐cell lung cancer (NSCLC), colon cancer, and renal cell carcinoma.^2^

At present, there are two main types of ICIs that have entered clinical applications. First, programmed cell death protein‐1 (PD‐1) is expressed on the T lymphocyte membrane and programmed cell death ligand‐1 (PD‐L1) is highly expressed in tumor cells. Activation of the PD‐1 signaling pathway impairs T cell function and their immune response. The PD‐1 antibodies (pembrolizumab and nivolumab) and the PD‐L1 antibodies (atezolizumab, avelumab and durvalumab) can prevent PD‐L1 binding to PD‐1, thus blocking this negative regulatory signaling pathway and enhancing the body's immune function against tumors. Another immune checkpoint molecule is cytotoxic T lymphocyte‐associated antigen 4 (CTLA‐4), which is also an inhibitory receptor on the surface of T cell membrane. Ipilimumab is a representative of CTLA‐4 inhibitors that activate T cell anti‐tumor responses by removing tumor‐induced immunosuppression.^2^ However, with the vigorous development of therapeutic drugs at tumor immune checkpoints, the side effects of these new drugs began to surface, and the clinical safety of these drugs was a concern. Even though the incidence of cardiac immune‐related adverse events (irAEs) was relatively low according to the literature,^3^ the diagnosis and management of ICI‐associated cardiotoxic effects are highly challenging.^4^ This review summarizes the latest evidence regarding the epidemiology of cardiotoxicity, as well as their clinical manifestation and management.

## Epidemiology

Many kinds of ICI‐associated cardiotoxicity have been reported including myocardial lesions (mainly myocarditis), pericardial effusion, arrhythmia, acute coronary syndrome, valvular disease, and systemic vasculitis. According to the VigiBase (the WHO's global database of individual case safety reports) in which 31 321 cardiac irAEs were reported in patients who received ICIs from 2008 to 2018, cardiac irAEs with a higher incidence than that of other drugs included myocarditis (0.39%), pericardial disease (0.30%), supraventricular tachycardia (0.71%) and vasculitis (temporal arteritis and rheumatic polymyalgia) (0.26%).^3^ In addition, myocardial infarction, cardiac death and hypertension were also reported with an incidence of 0.53%, 0.43% and 0.63%, respectively.^3^ With the growing awareness of the potential autoimmune side effects of ICIs among the oncology and cardiology communities, the incidence of ICI‐associated cardiovascular toxic effects appears to be underestimated, and the true incidence of cardiotoxic effects from ICIs needs to be re‐evaluated in the real‐world practice.

## Clinical manifestation

### ICI‐associated myocardial lesions

#### ICI‐associated myocarditis

ICI‐associated myositis is a severe irAE with a high mortality rate (39.7%–50%).^5^ The reported incidence rate in the study by Anquetil *et al*. was 0.06%–3.8%, and the median onset time was 18–39 days after the first dose.^6^ Often characterized by acute or fulminant attacks, myocarditis may quickly progress to severe heart failure, cardiogenic shock and even cardiac arrest within 1–2 weeks. The common clinical symptoms include dyspnea, chest pain, palpitations, fatigue, and myalgia. Mild to moderate elevation of serum troponin which does not meet the rise and fall pattern of acute myocardial infarction supports the diagnosis of myocarditis, although negative troponin and/or creatinine kinase levels does not exclude the diagnosis.^7^ The level of brain natriuretic peptide (BNP) or N‐terminal pro‐brain natriuretic peptide (NT‐proBNP) will progressively elevate; blood inflammatory indicators such as C‐reactive protein and erythrocyte sedimentation rate are significantly increased. Electrocardiogram (ECG) findings include ST‐T changes, Q wave formation, R wave decrease, and widened QRS waves, possibly combined with various arrhythmias from sinus tachycardia to ventricular fibrillation. Echocardiography is an important tool in evaluating structural and functional changes secondary to myocarditis. It reveals general or segmental wall motion reduction, no significant enlargement of cardiac chambers, and reduced left ventricular (possibly combined with right ventricular) ejection fraction, with or without moderate to severe valvular insufficiency. Coronary angiography should not show severe obstructive lesions. Cardiac enhanced MR (CMR), as the most useful noninvasive diagnostic modality for myocarditis, will find myocardial edema or delayed myocardial enhancement, as well as decreased left ventricular ejection fraction. Cardiac biopsy as the gold standard diagnostic modality usually reveals predominant lymphocytic infiltrates in the myocardium and conduction system. Immunohistochemistry shows CD3 +/CD8 + cells with the infiltration of a few CD4 + cells.^8^, ^9^ There are also a few cases reporting multinuclear giant cellular infiltration in the myocardium.^10^, ^11^

#### ICI‐associated cardiomyopathy

If patients receiving ICI treatment complain about fatigue, progressive dyspnea and chest pain, it is necessary to consider the diagnosis of dilated cardiomyopathy in addition to myocarditis. It is difficult to differentiate these according to clinical symptoms. Blood levels of cardiac troponin in these patients are more prone to be normal, but BNP or NT‐proBNP could be significantly elevated. The ECG will find QRS waveform changes, nonspecific ST‐T changes and various arrhythmias. Echocardiography shows segmental or diffused wall motion reduction, a normal or slight increase in left ventricle diameter and a reduction in the left ventricle (possibly combined with right ventricle) ejection fraction. Moderate to severe valvular insufficiency could also be found. Coronary angiography should not find severe obstructive lesions. CMR will show myocardial late gadolinium enhancement without significant myocardial edema. Endomyocardial biopsy revealed myocardial degeneration, necrosis, and interstitial fibrosis, but no obvious inflammatory cell infiltration.^10^ There are also case reports in which individual patients had no clinical signs of heart failure but were diagnosed with dilated cardiomyopathy by echocardiography or by autopsy.^10^

#### ICI‐associated stress cardiomyopathy

Stress cardiomyopathy is another type of cardiomyopathy associated with ICI therapy.^12^ Caution is required in differentiating this cardiomyopathy from myocarditis by clinical manifestations. Recurrent or persistent chest pain and dyspnea are common symptoms that can progress rapidly. ECG will reveal ST segment elevation or depression in multiple adjacent leads and T wave abnormalities, as well as a variety of atrial and ventricular arrhythmias and even cardiac arrest. The level of cardiac troponin could be normal or slightly elevated and the level of BNP or NT‐proBNP is often significantly increased. Typical echocardiography and left ventriculography, which are helpful in identifying the diagnosis, are systolic apical ballooning of the left ventricle, reflecting depressed mid‐ and apical segments with hyperkinesis of the basal wall. Coronary angiography will exclude severe obstructive lesions.^13^, ^14^ CMR could find myocardial edema but usually without myocardial late gadolinium enhancement.^15^ The prevalence of stress cardiomyopathy in ICI‐associated cardiotoxicity remains unclear.

### ICI‐associated pericardial lesions

Once patients receiving ICI therapy develop exertional dyspnea, pericardial lesions should also be considered, which present as pericarditis or pericardial effusion. The incidence of pericardial lesions is approximately 0.3% and the median onset time is 30 days after the first dose of ICI treatment.^3^ It is suspected that patients with cancer who receive ICIs following radiotherapy to the thoracic area might be more prone to pericardial diseases.^16^ The ECG could show low voltage of the QRS complex in the limb and chest leads. The findings on chest radiograph are variable; small to moderate effusions may not result in significant findings, while larger pericardial effusions typically present with an enlarged cardiac silhouette with clear lung fields. Echocardiography will help to confirm the diagnosis. Short‐term rapid pericardial effusion growth can cause cardiac tamponade. Pericardial lesions should be suspected especially when the low voltage in ECG and sinus tachycardia happen at the same time. Once ICI‐associated pericardial effusion causes hemodynamic abnormalities (cardiac tamponade), emergent pericardial puncture should be performed. However, the analysis of pericardial effusion will not find malignant cells but instead predominant lymphocytes.

### ICI‐associated arrhythmia

The highest incidence of ICI‐associated arrhythmias is supraventricular arrhythmias including sinus tachycardia, frequent atrial premature beats, atrial tachycardia, atrial flutter or even atrial fibrillation. Supraventricular arrhythmias are commonly associated with other system irAEs such as hypoxia, infection, various lung diseases, or thyroid dysfunction, although supraventricular arrhythmias could be part of the manifestation of myocarditis or other cardiac irAEs. However, ventricular arrhythmias with relatively low incidence such as frequent ventricular premature beats, nonsustained ventricular tachycardia, torsades de pointes, partial or complete atrioventricular block, and even cardiac arrest are mostly accompanied by myocardial lesions especially myocarditis. Clinically, if palpitations, dizziness and a loss of consciousness appear, repeated electrocardiogram or Holter monitoring should be performed immediately. Histopathological examination may reveal lymphocytic infiltration in the conduction system.^17^ These arrhythmias could be the direct cause of death of ICI‐associated cardiotoxicity, which may be alleviated after effective control.^18^, ^19^

### ICI‐associated myocardial ischemia

It has been reported that patients using ICI could present with stable angina or acute coronary syndrome, and the latter may even require emergent coronary intervention. The exact incidence is not clear. Animal experiments have observed that T lymphocytes infiltrate coronary endothelium and lipid plaques after ICI application, increasing plaque instability, which may be the pathophysiological basis of myocardial ischemia after ICI administration.^5^, ^20^

### ICI‐associated valvular dysfunction

Clinically, there have been cases reporting moderate to severe aortic, mitral and tricuspid regurgitation. Most of these lesions are accompanied by myocardial lesions (myocarditis or cardiomyopathy). It is speculated that valve insufficiency may result from the inflammatory changes in valves with T‐cell infiltration and the dysfunction of valve appendages due to myocardial lesions.^10^

## ICI application and cardiotoxicity

Even though myocarditis and pericarditis are both relatively common cardiac irAEs of ICIs, some studies have implied that they seldom occur at the same time.^3^ PD‐1/PD‐L1 inhibitors appear to be more prone to myocarditis and pericardial disease than CTLA‐4 inhibitors, while CTLA‐4 inhibitors induce more vasculitis such as temporal arteritis.^3^, ^21^ The occurrence of myocarditis induced by PD‐1/PD‐L1 inhibitors may be related to the initial higher dose. There is evidence that the combined use of two ICIs may increase the incidence of myocarditis which has a significantly higher mortality rate than the myocarditis induced by ICI monotherapy (66% vs. 44%).^3^ It is also worth noting that myocarditis would be likely to occur with other systemic lesions (42%), and the common accompanied systemic damage includes neuromuscular abnormalities (such as skeletal muscle myositis, myasthenia gravis, and Guillain‐Barre synthesis) and hepatitis.^4^, ^21^ Therefore, if patients using ICIs develop skeletal muscle weakness, myalgia, dysphagia, or diplopia, or the level of creatine kinase is significantly elevated, screening for indicators of myocardial damage, such as troponin, BNP, and electrocardiogram are indicated.

## Diagnosis of ICI‐associated cardiotoxicity

**Figure 1:**
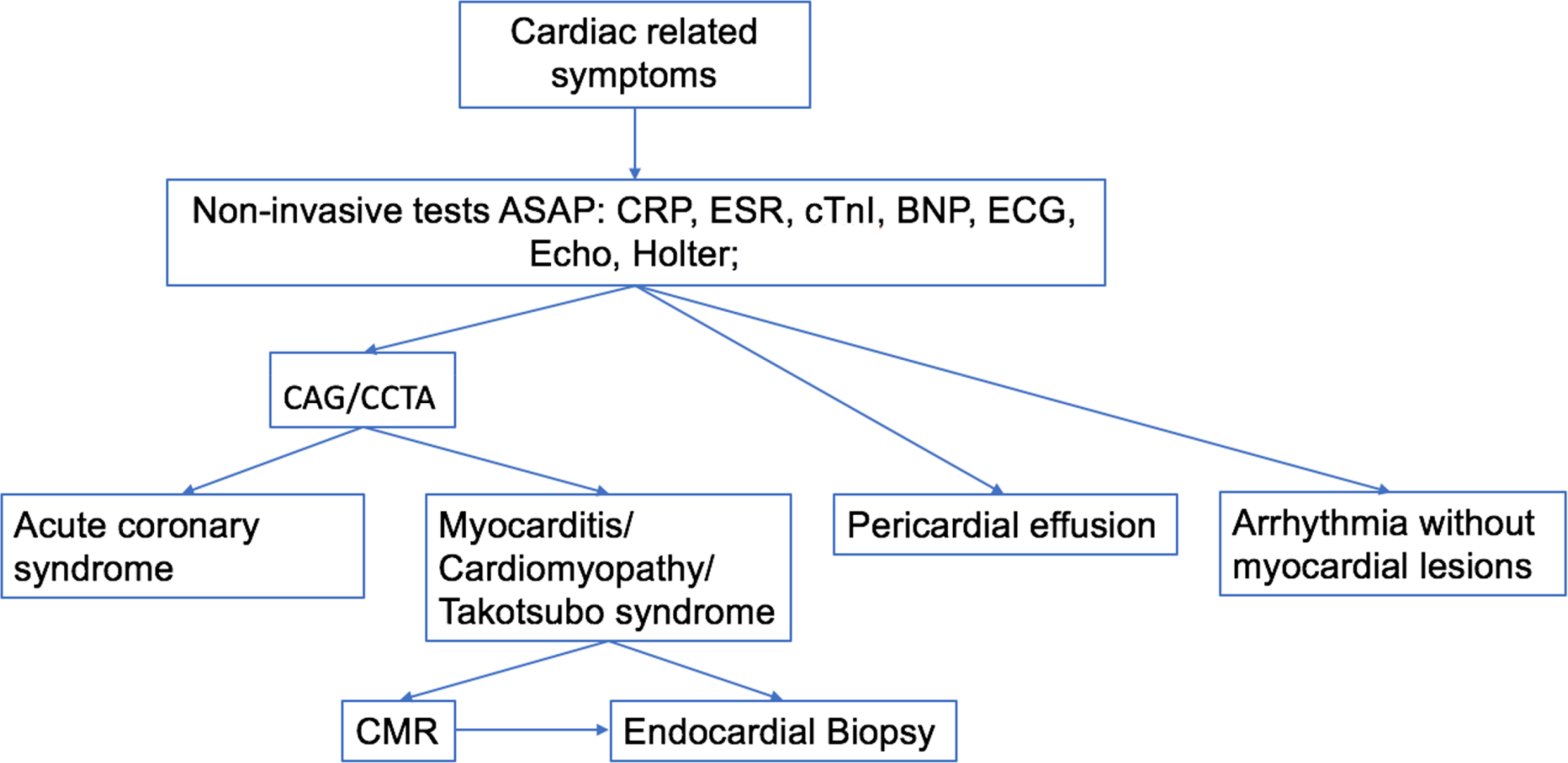
Flow chart of the diagnostic process of ICI‐associated cardiotoxicity.

Despite the low incidence of ICI‐associated cardiotoxicity, most incidences are life‐threatening. Thus, clinicians should be vigilant for signs of cardiac abnormalities at all times, and early diagnosis is important for improving the prognosis (Fig 1). Once clinical symptoms such as chest tightness, palpitations, and dyspnea are present, a noninvasive work‐up should be started as soon as possible, including blood tests of inflammation (erythrocyte sedimentation rate and C‐reactive protein), myocardial enzymes, BNP and an electrocardiogram.^22^ Of note, this diagnostic testing must be observed dynamically, which would be helpful in ruling out false positive results and evaluating the severity of the disease.

Echocardiography is indicated to be performed as the next step in patients suspected to have ICI‐associated cardiotoxicity based on symptoms and/or abnormal cardiac biomarkers. Diffused or regional ventricle wall motion, moderate to severe valve insufficiency or pericardial effusion support the presence of ICI‐associated cardiotoxicity, while in some cases, such as the early stage of myocarditis, the echocardiography reports may be negative.

If acute coronary syndrome is suspected according to the results of all these noninvasive tests, emergent coronary angiography or coronary enhancement computed tomography should be performed. CMR can offer more details in assessing the presence of myocardial inflammation and is recommended in all the patients in whom myocardial lesions are highly suspected. Myocardial edema and late gadolinium enhancement indicate the possibility of ICI‐associated myocarditis, though the absence of CMR signs could not exclude it.^23^ Unfortunately, some patients may not have a chance to undergo CMR testing due to the rapidly progressive and critical illness, and these patients' diagnosis will depend on bedside tests. Endomyocardial biopsy (EMB) using the Dallas Criteria remains the gold standard for the differential diagnosis of myocarditis and other myocardial lesions. Multidisciplinary teams, including cardiologists, oncologists and doctors in intensive care units are required to make the decision whether to undergo EMB, taking into consideration the potential benefits and risks of this procedure. There is no doubt that the presence or absence of characteristic lymphocytic infiltrates is valuable in guiding subsequent treatment and prognosis once the EMB is performed.^24^

## Treatment of ICI‐associated cardiotoxicity

The main strategy to treat ICI‐associated cardiotoxicity is to suppress the hyperactive T‐cell response. Because of the low incidence of ICI‐associated cardiotoxicity, current treatment recommendations are based on the treatment experience of the same non‐ICI‐associated diseases and noncardiac ICI‐associated toxicities. In addition to the immediate withdrawal of ICIs, glucocorticoid therapy is now the first‐line immunosuppressive treatment and may increase the survival chance in the setting of severe ICI‐associated cardiotoxicity. For hemodynamically stable patients with acute myocarditis or pericardial effusion, an initial dose of 1 mg/kg/day of intravenous methylprednisolone may be sufficient in the acute setting, followed by a slow oral prednisone taper over the course of a month or longer. For patients with life‐threatening conditions (fulminant myocarditis and/or malignant arrhythmia, pericarditis with cardiac tamponade), 1000 mg of intravenous methylprednisolone daily for 3–5 days followed by tapering to low‐dose prednisone should be considered. There are still some patients who do not respond well to glucocorticoids. Other immunosuppressive regimens have been proposed as adjunctive therapy. Plasmapheresis, intravenous immunoglobulins, antithymocyte globulin, mycophenolate mofetil, tacrolimus, methotrexate and infliximab have been tested in different cases, though the efficacy of these therapies is lacking.^21^

In addition to immunosuppressive therapy, effective supportive care and guideline‐directed management should be employed for patients. Those with heart failure should receive guideline‐directed medical therapy including beta‐blockers and a renin‐angiotensin II inhibitor/angiotensin receptor blocker. Those who develop tamponade, pericardiocentesis or pericardial window placement are necessary. Patients with life‐threatening arrhythmias should receive appropriate antiarrhythmic agents such as intravenous amiodarone and be considered for temporary and potentially permanent pacemaker placement as needed for advanced conduction disease. If the patient has persistent refractory heart failure, in addition to vasoactive drugs, IABP(intraaortic balloon pump) or ECMO (extracorporeal membrane oxygenation) support therapy might be attempted.^23^

## Early detection of ICI‐associated cardiotoxicity

There is currently no high‐quality study to investigate the characteristics of patients at high risk of developing ICI‐associated cardiotoxicity. However, the following groups may require special attention empirically: first, patients receiving the combination of two kinds of ICI drugs or the usage of ICI drugs combined with other antitumor drugs with cardiotoxicity (such as tyrosine kinase inhibitors)^25^; second, patients with a history of cardiac lesions caused by previous usage of antitumor drugs; and third, the patients with cardiac diseases (such as coronary heart disease, heart failure, myocarditis and valve diseases).^20^

Before administrating the ICIs, the patient history of heart diseases and medications should be carefully reviewed. If there is suspected myocardial ischemia, coronary evaluation (myocardial stress test, coronary CTA or coronary angiography) should be performed. If patients have a history of arrhythmias, Holter monitoring is suggested. The baseline tests including cTnI, NT‐proBNP/BNP, ECG, echocardiography, and inflammatory indicators are also needed.^26^ During ICI treatment at each visit, patients should be asked about cardiac‐related symptoms and receive careful physical examination. Noninvasive tests, including cTnI, NT‐proBNP/BNP and ECG, should be performed every 1–2 weeks in the first six weeks of treatment. If there is any new onset abnormality, it should be observed dynamically, and a cardiologist consultant is needed.

## Summary

ICI therapy has brought a novel and revolutionary solution to previously untreatable malignancies. However, this major advance is accompanied by a spectrum of cardiac side effects that are not common but are potentially fatal conditions. Additional research on the underlying mechanisms will offer new insights into irAEs. Clinical trials on patients presenting ICI‐associated cardiotoxicity are needed to elucidate the predisposing risk factors, identify the monitoring plan, and optimize the treatment strategy. All these investigations will be critical to provide further guidance for improving the medical care of patients receiving ICI therapy.

## Disclosure

The authors report no conflict of interest.
